# Ecological momentary assessment of fatigue, sleepiness, and exhaustion in ESKD

**DOI:** 10.1186/1471-2369-15-29

**Published:** 2014-02-06

**Authors:** Khaled Abdel-Kader, Manisha Jhamb, Lee Anne Mandich, Jonathan Yabes, Robert M Keene, Scott Beach, Daniel J Buysse, Mark L Unruh

**Affiliations:** 1 Division of Nephrology and Hypertension, Vanderbilt University Medical Center, 1161 21st Avenue South, MCN S-3223, Nashville, TN 37232-2372, USA; 2 Renal-Electrolyte Division, University of Pittsburgh, Pittsburgh, PA, USA; 3 Renal Section, VA Pittsburgh Healthcare System, Pittsburgh, PA, USA; 4 Center for Research on Health Care, Division of General Internal Medicine, University of Pittsburgh, Pittsburgh, PA, USA; 5 University Center for Social and Urban Research, University of Pittsburgh, Pittsburgh, PA, USA; 6 Department of Psychiatry, Sleep Medicine Institute, University of Pittsburgh, Pittsburgh, PA, USA; 7 Division of Nephrology, University of New Mexico, Albuquerque, NM, USA

**Keywords:** End-stage renal disease, Alertness, Fatigue, Symptoms, Ecological momentary assessment

## Abstract

**Background:**

Many patients on maintenance dialysis experience significant sleepiness and fatigue. However, the influence of the hemodialysis (HD) day and circadian rhythms on patients’ symptoms have not been well characterized. We sought to use ecological momentary assessment to evaluate day-to-day and diurnal variability of fatigue, sleepiness, exhaustion and related symptoms in thrice-weekly maintenance HD patients.

**Methods:**

Subjects used a modified cellular phone to access an interactive voice response system that administered the Daytime Insomnia Symptom Scale (DISS). The DISS assessed subjective vitality, mood, and alertness through 19 questions using 7- point Likert scales. Subjects completed the DISS 4 times daily for 7 consecutive days. Factor analysis was conducted and a mean composite score of fatigue-sleepiness-exhaustion was created. Linear mixed regression models (LMM) were used to examine the association of time of day, dialysis day and fatigue, sleepiness, and exhaustion composite scores.

**Results:**

The 55 participants completed 1,252 of 1,540 (81%) possible assessments over the 7 day period. Multiple symptoms related to mood (e.g., feeling sad, feeling tense), cognition (e.g., difficulty concentrating), and fatigue (e.g., exhaustion, feeling sleepy) demonstrated significant daily and diurnal variation, with higher overall symptom scores noted on hemodialysis days and later in the day. In factor analysis, 4 factors explained the majority of the observed variance for DISS symptoms. Fatigue, sleepiness, and exhaustion loaded onto the same factor and were highly intercorrelated. In LMM, mean composite fatigue-sleepiness-exhaustion scores were associated with dialysis day (coefficient and 95% confidence interval [CI] 0.21 [0.02 – 0.39]) and time of day (coefficient and 95% CI 0.33 [0.25 – 0.41]. Observed associations were minimally affected by adjustment for demographics and common confounders.

**Conclusions:**

Maintenance HD patients experience fatigue-sleepiness-exhaustion symptoms that demonstrate significant daily and diurnal variation. The variability in symptoms may contribute to poor symptom awareness by providers and greater misclassification bias of fatigue related symptoms in clinical studies.

## Background

Over 50% of patients on maintenance dialysis experience significant sleepiness and fatigue [1]. These symptoms affect dialysis patients’ quality of life and daily activities [2-4] and are independently associated with mortality and cardiovascular events [5,6]. Previous work revealed significant day-to-day variability in non-dialysis dependent chronic kidney disease (CKD) and end-stage kidney disease (ESKD) patient reported sleep quality, mood, alertness, and symptom burden [7,8]. However, the influence of the hemodialysis (HD) day and circadian rhythms on ESKD patients’ symptoms have not been well characterized.

One potential contributor to ESKD symptom variability is the HD procedure, which may impact patient activity patterns, sleep, arousal, thermoregulatory processes, and the uremic milieu with subsequent effects on sleepiness and fatigue [8-11]. Regardless of its cause, symptom variability may have important clinical and research implications including providing further insight into causal factors, ameliorative strategies, and limitations of recall-based symptom assessment methods. Despite these considerations, studies examining the daily and diurnal variation of sleepiness and fatigue in the maintenance dialysis population are scant.

One method that can be used to capture symptom variability is ecological momentary assessment (EMA). EMA is a tool to prospectively, repeatedly evaluate patient symptoms in real-time, in their natural environment, and while minimizing respondent burden [12]. Whereas conventional recall-based questionnaires may fail to depict the considerable day-to-day and diurnal variation in symptoms, EMA is well suited for this purpose [13,14]. We prospectively followed a cohort of maintenance HD patients for 1 week, using EMA to serially assess their symptom burden in real-time. We hypothesized *a priori* that fatigue, sleepiness and exhaustion would demonstrate significant diurnal variation [15,16] and symptoms would be more severe on dialysis days.

## Methods

### Participants, study setting, design

As part of a larger prospective cohort study investigating sleep, mood, and alertness [7], English-speaking patients undergoing thrice-weekly in-center maintenance hemodialysis were approached during their routine dialysis clinic visit or their initial evaluation at a kidney transplantation clinic between March 2008 and January 2010. As previously described [7], exclusion criteria included age <18 years or >90 years, use of continuous positive airway pressure, severe comorbid illness (e.g., unstable angina, active malignancy), and active psychiatric condition [17,18]. In addition, for this study, patients with nocturnal work schedules were excluded. The study was approved by the University of Pittsburgh institutional review board and complied with the Helsinki Declaration of 1975. All participants provided written informed consent.

For this study, 77 participants provided informed consent. Subsequently, 8 withdrew due to loss of interest, 4 were withdrawn by the investigators due to poor adherence with the current/parent study, 3 participants died, and 7 withdrew for other reasons (e.g., deteriorating health/hospitalization, severe auditory deficits). The remaining 55 maintenance dialysis patients are included in this report.

Following enrollment, participants chose a convenient week to complete the EMA. Participants used a modified cellular phone to access an interactive voice response system that administered the Daytime Insomnia Symptom Scale (DISS) [15]. The DISS assesses subjective fatigue/sleepiness, mood, and cognitive alertness through 19 short questions using a 7-point Likert scale (e.g., How fatigued do you feel right now? How sleepy do you feel right now?) with higher scores representing greater endorsement of a particular symptom. Participants completed the DISS 4 times daily for 7 consecutive days with each assessment generally requiring 2 to 6 minutes. Calls were performed at wake-up time, noon, 6 pm, and bedtime. For the noon and 6 pm measurements, automatic reminder calls occurred if a participant failed to call within 10 minutes of the pre-specified window. Through January 2010, 55 ESKD patients consented and completed 7 consecutive days of EMA.

### Data collection

Baseline data collection included a brief standardized health interview and questionnaire. Age, sex, race, education, employment, alcohol use, and smoking status were self-reported. A history of physician-diagnosed medical illnesses, previous surgical treatments, or medical procedures was obtained during the health interview. Standard serum laboratory tests drawn ≤ 3 months prior to or following EMA, were abstracted from the medical record.

### Statistical analysis

Baseline demographics, comorbidities, and laboratory values were described using means and standard deviations for continuous variables and frequencies and percentages for categorical variables. Mean scores were determined for each of the DISS items by day (i.e., HD vs. non-HD) and time of day (wake-up time, noon, 6 pm, bedtime). For consistency, several DISS items that assess positive mood (e.g., happy) and cognition (e.g., alert) were reverse coded for the analysis so that higher scores represented greater symptom burden (i.e., not happy). Exploratory factor analysis using principal components with oblimin rotation was conducted on the DISS items. The optimal coordinates method determined the number of factors retained. Factor loadings for fatigue, sleepiness, and exhaustion by day and time were determined. Because fatigue, sleepiness, and exhaustion loaded together (with factor loadings that were quantitatively very similar) on the same factor at all days and times and exhibited high correlation, for clarity, the mean fatigue-sleepiness-exhaustion (FSE) score was calculated for each day/time permutation and used in mixed model regression analyses. As sensitivity analyses, alternative factor rotations were examined and mixed model regression was repeated using the fatigue, sleepiness, and exhaustion factor loadings to determine scores for each day/time permutation.

Linear mixed regression models (LMM) with a random subject intercept and fixed time of day and dialysis day variables were used to examine the association of each symptom (dependent variable) with time of day and dialysis day while accounting for within-subject correlations across multiple time points and days. Based on graphical examination of the data, a quadratic term for time of day was introduced to model the possible curvilinear association between symptoms and time of day. The interaction between time of day and dialysis day was also tested.

Additional LMMs were constructed examining the association of composite FSE score and time of day, dialysis day while adjusting for demographics (i.e., age, sex, race) and individual covariates that were a priori identified as potentially influencing fatigue, sleepiness, and exhaustion [1]. The covariates were diabetes, cardiovascular disease, hemoglobin, albumin, phosphate, and dialysis adequacy (i.e., Kt/V). The 95% confidence intervals (CI) for the LMM coefficients were determined using Markov-Chain Monte Carlo simulations [19]. For all analyses, P values < 0.05 were considered significant. Analyses were performed in R 3.0.1 [20]. The packages psych, lme4, and languageR were used to conduct factor analysis, fit the LMMs, and provide inference for the LMM models, respectively [21-23].

## Results

The 55 study participants completed 1,252 of 1,540 (81%) possible assessments over the 7 day period, which did not differ by dialysis day. Baseline characteristics of the sample are shown in Table 1. The mean age was approximately 57 years with a modest predominance of men and a substantial number of African-Americans included. Over 90% of participants had achieved an education level of 12^th^ grade or higher. A large proportion of the sample had comorbidities. All participants achieved adequate Kt/V.

**Table 1:** Baseline characteristics

	**N (%) or mean (SD)**
Age (yr)	56.7 (17.3)
Men	33 (60)
Black	18 (32.7)
Employed/student	7 (12.7)
Education level grade 12 or above	51 (92.7)
Married	20 (36.4)
Diabetes	29 (52.7)
Hypertension	54 (98.2)
Cardiovascular disease	23 (41.8)
Active smoker	8 (14.5)
Alcohol use (any)	10 (18.2)
Hemoglobin (g/dL)	12.0 (1.3)
Albumin† (g/dL)	3.7 (0.5)
Creatinine (mg/dL)	7.7 (2.7)
BUN (mg/dL)	44.1 (14.9)
Phosphate†* (mg/dL)	5.1 [4.5, 5.8]
PTH‡ (pg/mL)	280 (175)
spKt/V**	1.6 (0.2)

Continuous variables are presented as means with (standard deviations). Categorical variables are expressed as frequencies and (percentages). N = 55 except as noted. †N = 50, *median, [25 percentile, 75 percentile], ‡N = 42, **N = 41.

BUN blood urea nitrogen, PTH parathyroid hormone.

**Figure 1:**
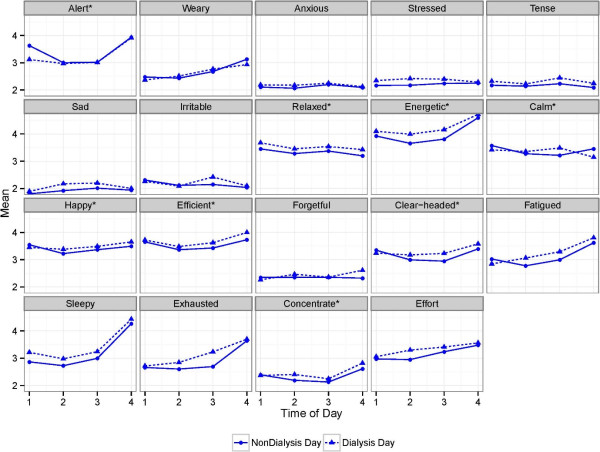
**Daily and diurnal symptom variation.** Higher scores indicate greater endorsement of listed symptom. Except as noted, items are of the form, “How _____ do you feel?” *Items were reverse coded so that higher scores would indicate greater symptom burden (e.g., “How happy do you feel?” was reverse coded to create “not happy”) Concentrate: actual wording is, “How well are you able to concentrate?” Effort: actual wording is, “How much of an effort is it to do anything?”.

The daily (non-HD vs. HD) and diurnal patterns of symptoms for each of the 19 DISS items are shown in Figure 1. In LMMs, while adjusting for the effect of time of day, significant differences between non-dialysis and HD day symptom scores were noted for: feeling sleepy, feeling exhausted, difficulty concentrating, feeling sad, not feeling relaxed, not feeling energetic, feeling stressed, and feeling tense (Additional file 1: Table S1). For each of these symptoms, significantly higher (i.e., worse) scores were associated with the dialysis day.

After adjusting for dialysis day, time of day was significantly associated with: feeling sleepy, feeling exhausted, difficulty concentrating, effort required to do things, feeling fatigued, not feeling energetic, not feeling alert, and feeling weary (Additional file 1: Table S1). The highest scores, indicating greater symptom burden, were generally observed later in the day. When modeling included a quadratic term for time of day to account for a possible curvilinear relationship, not feeling happy, not feeling efficient, not feeling clear headed, feeling sad, and feeling tense were also associated with time of day (Additional file 1: Table S1).

Exploratory factor analysis was undertaken on DISS items and 4 factors were retained. The 4 factors accounted for 77% of the variance in DISS items (Table 2). The DISS items and respective factor loadings are listed in Table 3. The first factor, labeled Negative Mood, included 6 items with loadings >0.4: anxious, stressed, tense, sad, irritable, and forgetful. The second factor, labeled Positive Mood, included 7 items with loadings > 0.4: alert, relaxed, energetic, calm, happy, efficient, and clear-headed. The third principal component, labeled Fatigue/Sleepiness, included 4 items with loadings > 0.4: weary, fatigued, sleepy, and exhausted. The fourth factor was difficult to characterize due to the lack of symptom items with strong loading weights, but was labeled alert cognition based on the items with the highest loading factors: forgetful, alert, clear-headed, and concentrate. Two symptom items did not load with a weight above 0.4 on any principal component: effort and concentrate. When alternative factor rotations were examined, DISS items exhibited stable and quantitatively similar factor loadings (Additional file 1: Table S2).

**Table 2:** Proportion of variance in DISS items explained by factors

	**Negative mood**	**Positive mood/cognition**	**Fatigue/sleepiness**	Alert cognition*
Variance explained	0.427	0.224	0.080	0.042
Cumulative variance explained	0.427	0.652	0.731	0.773

*Lacked strong factor loadings, but was characterized based on 4 items with largest factor loadings.

DISS daytime symptoms in insomnia scale.

**Table 3:** Factor loadings of DISS items

	**Negative mood**	**Positive mood**	**Fatigue/sleepiness**	Alert cognition*
Anxious	**0.92**	−0.01	−0.02	0.15
Stressed	**0.86**	0.04	0.12	−0.07
Tense	**0.90**	0.03	0.08	−0.14
Sad	**0.86**	−0.01	−0.03	−0.01
Irritable	**0.77**	0.07	0.15	0.00
Forgetful	**0.59**	0.01	0.19	0.19
Alert	−0.11	**0.85**	0.05	0.21
Relaxed	0.19	**0.82**	−0.12	−0.11
Energetic	−0.23	**0.77**	0.32	−0.12
Calm	0.19	**0.84**	−0.14	−0.06
Happy	0.11	**0.86**	−0.07	−0.12
Efficient	−0.08	**0.84**	0.14	−0.05
Clear-headed	0.01	**0.86**	−0.06	0.22
Weary	0.27	−0.05	**0.52**	0.14
Fatigued	0.11	−0.03	**0.81**	−0.01
Sleepy	0.04	0.00	**0.85**	−0.01
Exhausted	0.11	0.00	**0.88**	−0.01
Concentrate^†^	0.20	0.22	0.31	0.25
Effort^†^	0.08	0.23	0.31	0.12

^†^Except as noted, items are of the form, “How _____ do you feel?”.

*Lacked strong factor loadings, but was characterized based on 4 items with largest factor loadings.

Concentrate: actual wording is, “How well are you able to concentrate?”.

Effort: actual wording is, “How much of an effort is it to do anything?”.

DISS daytime symptoms in insomnia scale.

Bolded items denote primary factor loading.

Exploratory factor analysis stratified by day and time of day was also conducted. Fatigue, sleepiness, and exhaustion loaded onto the same factor at each time point and were highly correlated with factor loadings that demonstrated similar contributions from each of the individual symptom scores for each day and time of day (data not shown). Because fatigue, sleepiness, and exhaustion scores loaded onto the same factor together with comparable factor loading at each time point, and for the sake of clarity, the mean fatigue, sleepiness, and exhaustion composite score for each day and time of day was used for regression analyses.

**Figure 2:**
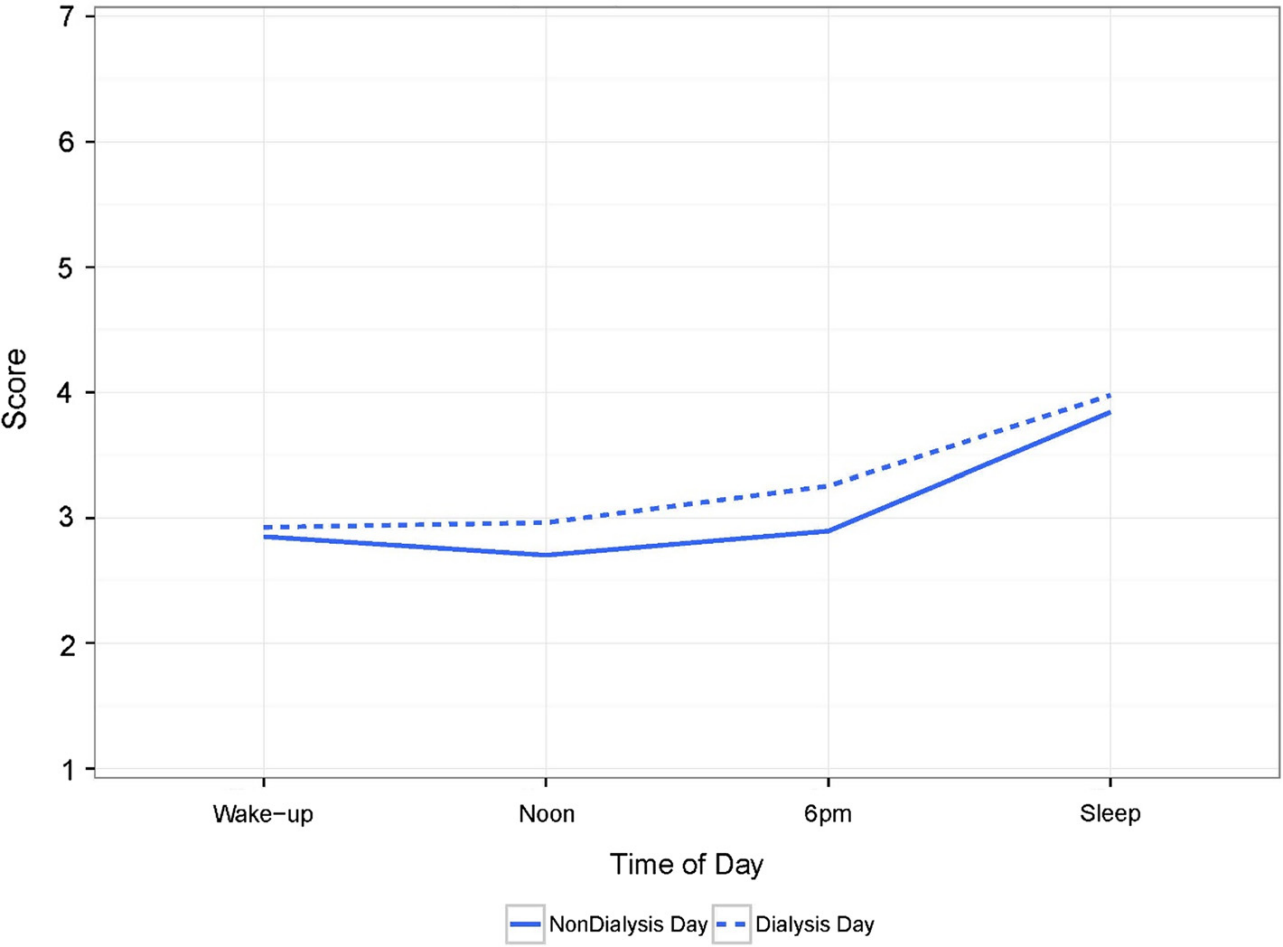
**Mean FSE composite scores.** FSE fatigue/sleepiness/exhaustion.

The mean FSE composite scores revealed that FSE scores increased later in the day and FSE scores were modestly higher on dialysis days (Figure 2). In LMM, mean composite FSE score was associated with time of day and dialysis day (Table 4). While controlling for dialysis day, each increase in time of day (e.g., from wake-up to noon or noon to 6 pm) increased FSE composite score by 0.33 (95% confidence interval [CI] 0.25-.41). While adjusting for time of day, dialysis day was associated with a 0.21 (95% CI 0.02-0.39) point increase in FSE composite score. Inclusion of a quadratic term to model a curvilinear relationship for time of day improved model performance but did not qualitatively change model interpretation (Additional file 1: Table S3). There was no modification of the effect of time of day by dialysis day.

**Table 4:** Adjusted association of FSE composite score with time of day and dialysis day

**Model**	**Variable**	**Coefficient estimate (95% CI)**	**P-value**
Unadjusted	Time of day	0.33 (0.25, 0.41)	<0.001
	Dialysis day	0.21 (0.02, 0.39)	0.03
Adjusted for demographics*	Age (per year)	−0.03 (−0.04, -0.01)	<0.001
	Sex (female vs. male)	−0.08 (−0.51, 0.39)	0.80
	Race (black vs. non-black)	−0.42 (−0.84, 0.09)	0.11
	Time of day	0.33 (0.25, 0.41)	<0.001
	Dialysis day	0.21 (0.03, 0.40)	0.03
Demographics + Kt/V	Age (per year)	−0.03 (−0.04, -0.01)	<0.001
	Sex (female vs. male)	−0.15 (−0.70, 0.42)	0.67
	Race (black vs. non-black)	−0.69 (−1.21, -0.07)	0.03
	Kt/V	−0.63 (−1.8, 0.56)	0.34
	Time of day	0.33 (0.23, 0.42)	<0.001
	Dialysis day	0.17 (−0.04, 0.38)	0.10

*Serial additional adjustment for diabetes, cardiovascular disease, albumin, hemoglobin, and phosphate did not affect time of day or dialysis day coefficients.

Adjusting the LMM for age, sex, and race did not alter the coefficient estimates for time of day or dialysis day (Table 4). Serial adjustments for demographics and individual covariates (i.e., hemoglobin, diabetes, cardiovascular disease, albumin, hemoglobin, and phosphate) also did not alter coefficient estimates. However, adjustment for demographics and Kt/V mildly attenuated the association between FSE score and dialysis day (Table 4).

As a sensitivity analysis, regression modeling was confirmed using scores derived from the fatigue, sleepiness, and exhaustion factor loadings as well as a quadratic term to model time of day (Additional file 1: Table S4).

## Discussion

In this study, maintenance HD patients exhibited high adherence to EMA for symptom documentation through 7 consecutive days. Our findings demonstrated significant diurnal and day-to-day variability of symptoms including fatigue, sleepiness, and exhaustion. Subsequent factor analysis identified 4 factors explaining most of the observed variance in the 19 symptom scores. Among these symptoms, fatigue, sleepiness, and exhaustion scores were highly intercorrelated and loaded onto the same factor for each time of day/dialysis day permutation. After controlling for potential confounders, composite FSE scores were associated with time of day and dialysis day. We observed an approximate 1 point increase in FSE symptom score associated with progression from wake-up time to bedtime (i.e., a 3 unit increase in time of day) and a 0.21 point increase in FSE score on dialysis days. In general, FSE scores on dialysis days and non-dialysis day were similar at wake-up and bedtime; however, larger differences were apparent in the afternoon and early evenings.

Despite the ongoing interest in quality of life, mood and symptom related research, few studies in nephrology have employed EMA [24]. EMA offers a number of possible advantages in these settings. When conducted electronically, it allows investigators to more accurately assess adherence and prevent participants from spuriously completing serial measurements. The high rates of participant EMA adherence in the present study are consistent with the published literature [25-28]. This should offer investigators who wish to employ EMA methods reassurance regarding respondent burden.

Because EMA is well suited to identifying those who develop symptoms in a particular context, it could prove useful for recognizing patients who develop post-dialysis fatigue [29,30], a debilitating, prevalent, but understudied process. In this setting, EMA may generate more valid assessments of symptom severity than current recall methods [31,32] and facilitate further investigation of correlates, causes, and outcomes of post-dialysis fatigue. Additional advantages of EMA are the ability to repeatedly assess dynamic states in real-time and in the participant’s usual environment. Static or retrospective assessments of mood, quality of life, and symptoms are subject to recall bias and can be influenced by the participant’s current mood, state, and symptoms [33,34]. EMA allows for a more accurate assessment of these mutable states and may be particularly relevant in ESKD where complex treatments are likely to represent physiologic and psychological stressors.

In our study, we noted significant diurnal variation in 13 of 19 DISS symptoms as well as the FSE scale. We also noted small but significant increases in symptom burden on dialysis days. This included symptoms related to mood (e.g., feeling sad, feeling tense), cognition (e.g., difficulty concentrating), and fatigue (e.g., feeling exhausted, feeling sleepy). These findings complement prior data indicating maintenance dialysis patients have greater day-to-day variability in mood and alertness [7] and greater subjective well-being on non-dialysis days [35]. Additional studies comparing retrospective assessments and EMA of mood and symptoms in patients with ESKD are needed to further understand the clinical and research limitations of traditional measurement approaches in this population. Such research could clarify whether retrospective instruments contribute to poor provider awareness of ESKD patients’ symptoms in clinical settings [36] due to a failure to capture the severity of symptoms at time points when the patient is unlikely to interact with providers or complete questionnaires (e.g., 6 pm and bedtime).

Analogously, higher rates of misclassification of symptom or mood status could lead to bias in clinical studies [7]. For instance, in studies examining symptoms and outcomes in ESKD, misclassifying participants as symptom free would make the comparison groups more similar with respect to the exposure of interest. This misclassification could limit power to detect true differences in outcomes between exposure groups [37]. Given existing challenges in conducting adequately powered clinical research studies in nephrology, the use of measures that do not optimally capture a patient’s mood or symptoms could represent a readily correctable weakness.

Previous work identified 4 principal components for the DISS: negative mood, positive mood, alert cognition, and sleepiness/fatigue [15]. In factor analysis, we similarly identified 4 factors including negative mood, positive mood, sleepiness/fatigue, and alert cognition that explained a large proportion of the variance observed in the 19 symptom scores across days and time. We also noted that fatigue, sleepiness, and exhaustion were highly intercorrelated, corroborating earlier findings [15]. Although, composite FSE scores varied according to dialysis day, differences were larger at noon and 6 pm than they were at wake-up and bedtime (Figure 2).

These results appear to support previous findings indicating that nearly 75% of HD patients recover from the dialysis procedure by bedtime [38]. The timing also suggests aspects of the dialysis process may be causing the increase in fatigue, sleepiness, and exhaustion and may be indicative of post-dialysis fatigue. Disturbances to arousal and thermoregulatory mechanisms [9,10], ultrafiltration, changes in blood pressure, blood-membrane interaction [29], activation of inflammatory pathways [39], and/or psychological stressors [1,35] due to the procedure could explain these trends. EMA may be an important tool in future studies to identify and carefully examine how temporal patterns of fatigue impact on patient activities including exercise participation [40].

Although the modest 0.2 point increase in FSE scores associated with dialysis days in adjusted analyses is of uncertain clinical significance, it represents approximately 20% of the increase in FSE score associated with progression from wake-up to bedtime. In addition, multiple studies document the severe baseline fatigue burden in maintenance HD patients [1,6,35,41]. For example, Song and colleagues noted that separate from sleep hours, patients on maintenance HD rest for 5–6 hours on non-dialysis days [35]. Arguably, small increases above this baseline burden could be meaningful to patients and the promise of alleviating fatigue in ESKD is a potent patient motivator [42]. In addition, prior studies have shown that limited physical activity in ESKD patients is associated with increased mortality [43] and post-dialysis fatigue represents the most common barrier to exercise participation [40].

Our findings should be considered in light of several limitations. While our sample was relatively diverse and included 28 serial measurements per individual, larger studies are needed to corroborate our findings. Second, we did not ask subjects to record the precise time of HD treatments. Future studies using EMA in ESKD should consider collecting this information to further clarify the impact of the dialysis shift and procedure on mood, sleep, and symptoms. In addition, we used EMA to sample symptoms at fixed intervals throughout the day; using a large cohort and sampling at random intervals could reveal additional patterns of symptom variation. We also did not examine possible seasonal effects in daily and diurnal symptom variation. Finally, our study excluded institutionalized and home HD patients. Hence, our findings may not be generalizable to these populations.

## Conclusions

ESKD patients experience multiple symptoms including fatigue, sleepiness, and exhaustion that vary by dialysis day and time of day. FSE is greater on dialysis days and as expected, at bedtime. Our study demonstrated that maintenance dialysis patients can use EMA with high rates of adherence to document symptoms and mood to better understand variations and contextual associations. Additional studies are necessary to further characterize temporal factors that contribute to symptoms and mood disturbances in dialysis patients and their clinical and research implications.

## Abbreviations

CI: Confidence interval; CKD: Chronic kidney disease; DISS: Daytime insomnia symptom scale; EMA: Ecological momentary assessment; ESKD: End-stage kidney disease; FSE: Fatigue-sleepiness-exhaustion; HD: Hemodialysis; LMM: Linear mixed regression models.

## Competing interests

The authors declare that they have no competing interests.

## Authors’ contributions

KA, MJ, DB, SB, MU conceptualized the study. DB, MU obtained funding. KA, MJ, LAM, JY, RK, SB, and MU acquired the data. KA, MJ, JY, DB, MU analyzed the data. KA, MJ, LAM, JY, RK, SB, DB, MU contributed to interpretation and manuscript preparation. All authors read and approved the final manuscript.

## Pre-publication history

The pre-publication history for this paper can be accessed here:

http://www.biomedcentral.com/1471-2369/15/29/prepub

## Supplementary Material

Additional file 1 **Supplementary analyses. Table S1:** Linear mixed regression models for each DISS symptom, **Table S2:** Factor loadings from alternative factor rotations, **Table S3:** FSE Associations using LMM with quadratic term, **Table S4:** Adjusted association of factor analysis derived FSE score. Click here for file
