# Supplementary material for: Ecological momentary assessment of fatigue, sleepiness, and exhaustion in ESKD
Source: BMC Nephrol. 2014 Feb 6;15:29. doi: 10.1186/1471-2369-15-29 (PMC3927224; doi:10.1186/1471-2369-15-29)
Supplement: Additional file 1 — Supplementary analyses. Table S1: Linear mixed regression models for each DISS symptom, Table S2: Factor loadings from alternative factor rotations, Table S3: FSE Associations using LMM with quadratic term, Table S4: Adjusted association of factor analysis derived FSE score. [file 1471-2369-15-29-S1.doc]

**Appendix**

Table of contents

eTables 1: pages 2-11

eTables 2: pages 12-21

eTable 3: page 22

eTable 4: page 22

Abbreviations: TOD time of day, DD dialysis day, CI confidence interval

**eTables 1**. Linear mixed regression models for each of the DISS symptoms

Abbreviations: TOD time of day, DD dialysis day

**SLEEPY:**

| Model | Variable | Coefficient estimate | P-value |
| --- | --- | --- | --- |
| TOD, DD | Time of day | 0.43 | <0.0001 |
|  | Dialysis day | 0.25 | 0.02 |
| TOD, TOD squared, DD | Time of day | -1.41 | <0.0001 |
|  | Time of day squared | 0.37 | <0.0001 |
|  | Dialysis day | 0.25 | 0.01 |
| TOD, TOD squared, DD, TOD * DD interaction term | Time of day | -1.39 | <0.0001 |
|  | Time of day squared | 0.37 | <0.0001 |
|  | Dialysis day | 0.40 | 0.09 |
|  | Time of day*dialysis day | -0.06 | 0.48 |
| TOD, TOD squared, DD, TOD * DD interaction term, TOD * DD squared interaction term | Time of day | -1.39 | <0.0001 |
|  | Time of day squared | 0.37 | <0.0001 |
|  | Dialysis day | 0.40 | 0.46 |
|  | Time of day*dialysis day | -0.06 | 0.91 |
|  | Time of day squared*dialysis day | -0.001 | 0.99 |

EXHAUSTED

| Model | Variable | Coefficient estimate | P-value |
| --- | --- | --- | --- |
| TOD, DD | Time of day | 0.32 | <0.0001 |
|  | Dialysis day | 0.21 | 0.01 |
| TOD, TOD squared, DD | Time of day | -0.58 | 0.01 |
|  | Time of day squared | 0.18 | <0.0001 |
|  | Dialysis day | 0.21 | 0.007 |
| TOD, TOD squared, DD, TOD * DD interaction term | Time of day | -0.59 | 0.02 |
|  | Time of day squared | 0.18 | <0.0001 |
|  | Dialysis day | 0.14 | 0.006 |
|  | Time of day*dialysis day | 0.03 | 0.68 |
| TOD, TOD squared, DD, TOD * DD interaction term, TOD * DD squared interaction term | Time of day | -1.00 | 0.001 |
|  | Time of day squared | 0.26 | <0.0001 |
|  | Dialysis day | -0.70 | 0.09 |
|  | Time of day*dialysis day | 0.86 | 0.03 |
|  | Time of day squared*dialysis day | -0.17 | 0.03 |

**NOT CONCENTRATE**

| Model | Variable | Coefficient estimate | P-value |
| --- | --- | --- | --- |
| TOD, DD | Time of day | 0.08 | 0.01 |
|  | Dialysis day | 0.14 | 0.04 |
| TOD, TOD squared, DD | Time of day | -0.65 | <0.0001 |
|  | Time of day squared | 0.15 | <0.0001 |
|  | Dialysis day | 0.14 | 0.03 |
| TOD, TOD squared, DD, TOD * DD interaction term | Time of day | -0.68 | <0.0001 |
|  | Time of day squared | 0.15 | <0.0001 |
|  | Dialysis day | 0.02 | 0.90 |
|  | Time of day*dialysis day | 0.05 | 0.99 |
| TOD, TOD squared, DD, TOD * DD interaction term, TOD * DD squared interaction term | Time of day | -0.81 | 0.001 |
|  | Time of day squared | 0.17 | <0.0001 |
|  | Dialysis day | -0.25 | 0.29 |
|  | Time of day*dialysis day | 0.32 | 0.34 |
|  | Time of day squared*dialysis day | -0.06 | 0.24 |

**EFFORT**

| Model | Variable | Coefficient estimate | P-value |
| --- | --- | --- | --- |
| TOD, DD | Time of day | 0.16 | 0.01 |
|  | Dialysis day | 0.15 | 0.09 |
| TOD, TOD squared, DD | Time of day | 0.05 | 0.85 |
|  | Time of day squared | 0.02 | 0.65 |
|  | Dialysis day | 0.15 | 0.08 |
| TOD, TOD squared, DD, TOD * DD interaction term | Time of day | 0.05 | 0.83 |
|  | Time of day squared | 0.02 | 0.64 |
|  | Dialysis day | 0.19 | 0.37 |
|  | Time of day*dialysis day | -0.02 | 0.84 |
| TOD, TOD squared, DD, TOD * DD interaction term, TOD * DD squared interaction term | Time of day | -0.15 | 0.65 |
|  | Time of day squared | 0.06 | 0.33 |
|  | Dialysis day | -0.22 | 0.64 |
|  | Time of day*dialysis day | 0.40 | 0.36 |
|  | Time of day squared*dialysis day | -0.08 | 0.34 |

**NOT HAPPY**

| Model | Variable | Coefficient estimate | P-value |
| --- | --- | --- | --- |
| TOD, DD | Time of day | 0.03 | 0.49 |
|  | Dialysis day | 0.13 | 0.07 |
| TOD, TOD squared, DD | Time of day | -0.36 | 0.04 |
|  | Time of day squared | 0.08 | 0.03 |
|  | Dialysis day | 0.13 | 0.07 |
| TOD, TOD squared, DD, TOD * DD interaction term | Time of day | -0.38 | 0.04 |
|  | Time of day squared | 0.08 | 0.03 |
|  | Dialysis day | 0.02 | 0.93 |
|  | Time of day*dialysis day | 0.04 | 0.48 |
| TOD, TOD squared, DD, TOD * DD interaction term, TOD * DD squared interaction term | Time of day | -0.55 | 0.03 |
|  | Time of day squared | 0.11 | 0.02 |
|  | Dialysis day | -0.33 | 0.40 |
|  | Time of day*dialysis day | 0.39 | 0.27 |
|  | Time of day squared*dialysis day | -0.07 | 0.32 |

**NOT EFFICIENT**

| Model | Variable | Coefficient estimate | P-value |
| --- | --- | --- | --- |
| TOD, DD | Time of day | 0.06 | 0.12 |
|  | Dialysis day | 0.15 | 0.07 |
| TOD, TOD squared, DD | Time of day | -0.72 | 0.02 |
|  | Time of day squared | 0.16 | 0.003 |
|  | Dialysis day | 0.14 | 0.05 |
| TOD, TOD squared, DD, TOD * DD interaction term | Time of day | -0.75 | 0.005 |
|  | Time of day squared | 0.16 | 0.003 |
|  | Dialysis day | -0.01 | 0.94 |
|  | Time of day*dialysis day | 0.06 | 0.34 |
| TOD, TOD squared, DD, TOD * DD interaction term, TOD * DD squared interaction term | Time of day | -0.72 | 0.02 |
|  | Time of day squared | 0.15 | 0.02 |
|  | Dialysis day | 0.04 | 0.93 |
|  | Time of day*dialysis day | 0.01 | 0.98 |
|  | Time of day squared*dialysis day | 0.01 | 0.88 |

**FORGETFUL**

| Model | Variable | Coefficient estimate | P-value |
| --- | --- | --- | --- |
| TOD, DD | Time of day | 0.05 | 0.20 |
|  | Dialysis day | 0.06 | 0.44 |
| TOD, TOD squared, DD | Time of day | 0.005 | 0.98 |
|  | Time of day squared | 0.008 | 0.87 |
|  | Dialysis day | 0.06 | 0.42 |
| TOD, TOD squared, DD, TOD * DD interaction term | Time of day | -0.04 | 0.87 |
|  | Time of day squared | 0.007 | 0.89 |
|  | Dialysis day | -0.20 | 0.28 |
|  | Time of day*dialysis day | 0.10 | 0.12 |
| TOD, TOD squared, DD, TOD * DD interaction term, TOD * DD squared interaction term | Time of day | 0.04 | 0.91 |
|  | Time of day squared | -0.008 | 0.89 |
|  | Dialysis day | -0.04 | 0.92 |
|  | Time of day*dialysis day | -0.06 | 0.88 |
|  | Time of day squared*dialysis day | 0.03 | 0.67 |

NOT CLEAR HEADED

| Model | Variable | Coefficient estimate | P-value |
| --- | --- | --- | --- |
| TOD, DD | Time of day | 0.04 | 0.27 |
|  | Dialysis day | 0.13 | 0.12 |
| TOD, TOD squared, DD | Time of day | -0.69 | 0.002 |
|  | Time of day squared | 0.15 | 0.001 |
|  | Dialysis day | 0.13 | 0.11 |
| TOD, TOD squared, DD, TOD * DD interaction term | Time of day | -0.73 | 0.001 |
|  | Time of day squared | 0.15 | 0.001 |
|  | Dialysis day | -0.10 | 0.61 |
|  | Time of day*dialysis day | 0.09 | 0.20 |
| TOD, TOD squared, DD, TOD * DD interaction term, TOD * DD squared interaction term | Time of day | -0.99 | 0.001 |
|  | Time of day squared | 0.20 | 0.001 |
|  | Dialysis day | -0.62 | 0.17 |
|  | Time of day*dialysis day | 0.61 | 0.14 |
|  | Time of day squared*dialysis day | -0.10 | 0.21 |

**FATIGUED**

| Model | Variable | Coefficient estimate | P-value |
| --- | --- | --- | --- |
| TOD, DD | Time of day | 0.26 | <0.0001 |
|  | Dialysis day | 0.16 | 0.08 |
| TOD, TOD squared, DD | Time of day | -0.50 | 0.10 |
|  | Time of day squared | 0.15 | 0.01 |
|  | Dialysis day | 0.15 | 0.06 |
| TOD, TOD squared, DD, TOD * DD interaction term | Time of day | -0.54 | 0.08 |
|  | Time of day squared | 0.15 | 0.01 |
|  | Dialysis day | -0.09 | 0.64 |
|  | Time of day*dialysis day | 0.10 | 0.18 |
| TOD, TOD squared, DD, TOD * DD interaction term, TOD * DD squared interaction term | Time of day | -0.92 | 0.01 |
|  | Time of day squared | 0.23 | 0.002 |
|  | Dialysis day | -0.87 | 0.05 |
|  | Time of day*dialysis day | 0.88 | 0.03 |
|  | Time of day squared*dialysis day | -0.16 | 0.06 |

**SAD**

| Model | Variable | Coefficient estimate | P-value |
| --- | --- | --- | --- |
| TOD, DD | Time of day | 0.04 | 0.24 |
|  | Dialysis day | 0.13 | 0.048 |
| TOD, TOD squared, DD | Time of day | 0.44 | 0.02 |
|  | Time of day squared | -0.08 | 0.03 |
|  | Dialysis day | 0.13 | 0.04 |
| TOD, TOD squared, DD, TOD * DD interaction term | Time of day | 0.44 | 0.02 |
|  | Time of day squared | -0.08 | 0.03 |
|  | Dialysis day | 0.15 | 0.33 |
|  | Time of day*dialysis day | -0.008 | 0.88 |
| TOD, TOD squared, DD, TOD * DD interaction term, TOD * DD squared interaction term | Time of day | 0.29 | 0.24 |
|  | Time of day squared | -0.05 | 0.30 |
|  | Dialysis day | -0.15 | 0.67 |
|  | Time of day*dialysis day | 0.30 | 0.36 |
|  | Time of day squared*dialysis day | -0.06 | 0.34 |

**IRRITABLE**

| Model | Variable | Coefficient estimate | P-value |
| --- | --- | --- | --- |
| TOD, DD | Time of day | -0.06 | 0.13 |
|  | Dialysis day | 0.07 | 0.26 |
| TOD, TOD squared, DD | Time of day | -0.01 | 0.94 |
|  | Time of day squared | -0.009 | 0.77 |
|  | Dialysis day | 0.07 | 0.27 |
| TOD, TOD squared, DD, TOD * DD interaction term | Time of day | -0.04 | 0.82 |
|  | Time of day squared | -0.009 | 0.76 |
|  | Dialysis day | -0.06 | 0.66 |
|  | Time of day*dialysis day | 0.05 | 0.33 |
| TOD, TOD squared, DD, TOD * DD interaction term, TOD * DD squared interaction term | Time of day | -0.18 | 0.39 |
|  | Time of day squared | 0.02 | 0.62 |
|  | Dialysis day | -0.36 | 0.27 |
|  | Time of day*dialysis day | 0.35 | 0.24 |
|  | Time of day squared*dialysis day | -0.06 | 0.31 |

**NOT RELAXED**

| Model | Variable | Coefficient estimate | P-value |
| --- | --- | --- | --- |
| TOD, DD | Time of day | -0.08 | 0.05 |
|  | Dialysis day | 0.19 | 0.03 |
| TOD, TOD squared, DD | Time of day | -0.10 | 0.66 |
|  | Time of day squared | 0.004 | 0.94 |
|  | Dialysis day | 0.19 | 0.03 |
| TOD, TOD squared, DD, TOD * DD interaction term | Time of day | -0.10 | 0.67 |
|  | Time of day squared | 0.004 | 0.94 |
|  | Dialysis day | 0.19 | 0.36 |
|  | Time of day*dialysis day | -0.0002 | 0.99 |
| TOD, TOD squared, DD, TOD * DD interaction term, TOD * DD squared interaction term | Time of day | -0.04 | 0.89 |
|  | Time of day squared | -0.008 | 0.90 |
|  | Dialysis day | 0.31 | 0.52 |
|  | Time of day*dialysis day | -0.11 | 0.79 |
|  | Time of day squared*dialysis day | 0.02 | 0.79 |

**NOT ENERGETIC**

| Model | Variable | Coefficient estimate | P-value |
| --- | --- | --- | --- |
| TOD, DD | Time of day | 0.21 | <0.0001 |
|  | Dialysis day | 0.25 | 0.003 |
| TOD, TOD squared, DD | Time of day | -0.86 | 0.001 |
|  | Time of day squared | 0.22 | <0.0001 |
|  | Dialysis day | 0.25 | 0.001 |
| TOD, TOD squared, DD, TOD * DD interaction term | Time of day | -0.86 | 0.001 |
|  | Time of day squared | 0.22 | <0.0001 |
|  | Dialysis day | 0.29 | 0.12 |
|  | Time of day*dialysis day | -0.02 | 0.80 |
| TOD, TOD squared, DD, TOD * DD interaction term, TOD * DD squared interaction term | Time of day | -1.13 | <0.0001 |
|  | Time of day squared | 0.27 | <0.0001 |
|  | Dialysis day | -0.28 | 0.52 |
|  | Time of day*dialysis day | 0.55 | 0.16 |
|  | Time of day squared*dialysis day | -0.11 | 0.14 |

**NOT CALM**

| Model | Variable | Coefficient estimate | P-value |
| --- | --- | --- | --- |
| TOD, DD | Time of day | -0.06 | 0.12 |
|  | Dialysis day | -0.03 | 0.74 |
| TOD, TOD squared, DD | Time of day | -0.22 | 0.31 |
|  | Time of day squared | 0.03 | 0.99 |
|  | Dialysis day | -0.03 | 0.74 |
| TOD, TOD squared, DD, TOD * DD interaction term | Time of day | -0.21 | 0.35 |
|  | Time of day squared | 0.03 | 0.46 |
|  | Dialysis day | 0.05 | 0.81 |
|  | Time of day*dialysis day | -0.03 | 0.68 |
| TOD, TOD squared, DD, TOD * DD interaction term, TOD * DD squared interaction term | Time of day | -0.69 | 0.02 |
|  | Time of day squared | 0.13 | 0.03 |
|  | Dialysis day | -0.92 | 0.05 |
|  | Time of day*dialysis day | 0.94 | 0.03 |
|  | Time of day squared*dialysis day | -0.19 | 0.02 |

**NOT ALERT**

| Model | Variable | Coefficient estimate | P-value |
| --- | --- | --- | --- |
| TOD, DD | Time of day | 0.16 | 0.001 |
|  | Dialysis day | -0.12 | 0.17 |
| TOD, TOD squared, DD | Time of day | -1.45 | <0.0001 |
|  | Time of day squared | 0.32 | <0.0001 |
|  | Dialysis day | -0.13 | 0.11 |
| TOD, TOD squared, DD, TOD * DD interaction term | Time of day | -1.52 | <0.0001 |
|  | Time of day squared | 0.32 | <0.0001 |
|  | Dialysis day | -0.48 | 0.01 |
|  | Time of day*dialysis day | 0.14 | 0.05 |
| TOD, TOD squared, DD, TOD * DD interaction term, TOD * DD squared interaction term | Time of day | -1.81 | <0.0001 |
|  | Time of day squared | 0.38 | <0.0001 |
|  | Dialysis day | -1.07 | 0.01 |
|  | Time of day*dialysis day | 0.74 | 0.06 |
|  | Time of day squared*dialysis day | -0.12 | 0.13 |

**WEARY**

| Model | Variable | Coefficient estimate | P-value |
| --- | --- | --- | --- |
| TOD, DD | Time of day | 0.21 | <0.0001 |
|  | Dialysis day | -0.03 | 0.73 |
| TOD, TOD squared, DD | Time of day | -0.13 | 0.54 |
|  | Time of day squared | 0.07 | 0.12 |
|  | Dialysis day | -0.03 | 0.74 |
| TOD, TOD squared, DD, TOD * DD interaction term | Time of day | -0.12 | 0.58 |
|  | Time of day squared | 0.07 | 0.12 |
|  | Dialysis day | 0.04 | 0.83 |
|  | Time of day*dialysis day | -0.03 | 0.70 |
| TOD, TOD squared, DD, TOD * DD interaction term, TOD * DD squared interaction term | Time of day | -0.42 | 0.16 |
|  | Time of day squared | 0.13 | 0.03 |
|  | Dialysis day | -0.57 | 0.22 |
|  | Time of day*dialysis day | 0.58 | 0.17 |
|  | Time of day squared*dialysis day | -0.12 | 0.14 |

**ANXIOUS**

| Model | Variable | Coefficient estimate | P-value |
| --- | --- | --- | --- |
| TOD, DD | Time of day | -0.007 | 0.83 |
|  | Dialysis day | 0.06 | 0.30 |
| TOD, TOD squared, DD | Time of day | 0.11 | 0.48 |
|  | Time of day squared | -0.02 | 0.44 |
|  | Dialysis day | 0.06 | 0.30 |
| TOD, TOD squared, DD, TOD * DD interaction term | Time of day | 0.12 | 0.44 |
|  | Time of day squared | -0.02 | 0.44 |
|  | Dialysis day | 0.13 | 0.15 |
|  | Time of day*dialysis day | -0.03 | 0.61 |
| TOD, TOD squared, DD, TOD * DD interaction term, TOD * DD squared interaction term | Time of day | 0.09 | 0.68 |
|  | Time of day squared | -0.02 | 0.70 |
|  | Dialysis day | 0.06 | 0.85 |
|  | Time of day*dialysis day | 0.04 | 0.89 |
|  | Time of day squared*dialysis day | -0.01 | 0.82 |

**STRESSED**

| Model | Variable | Coefficient estimate | P-value |
| --- | --- | --- | --- |
| TOD, DD | Time of day | -0.002 | 0.95 |
|  | Dialysis day | 0.14 | 0.04 |
| TOD, TOD squared, DD | Time of day | 0.12 | 0.57 |
|  | Time of day squared | -0.02 | 0.53 |
|  | Dialysis day | 0.14 | 0.04 |
| TOD, TOD squared, DD, TOD * DD interaction term | Time of day | 0.14 | 0.50 |
|  | Time of day squared | -0.02 | 0.54 |
|  | Dialysis day | 0.28 | 0.10 |
|  | Time of day*dialysis day | -0.05 | 0.39 |
| TOD, TOD squared, DD, TOD * DD interaction term, TOD * DD squared interaction term | Time of day | 0.04 | 0.89 |
|  | Time of day squared | -0.003 | 0.95 |
|  | Dialysis day | 0.06 | 0.87 |
|  | Time of day*dialysis day | 0.16 | 0.65 |
|  | Time of day squared*dialysis day | -0.04 | 0.54 |

**TENSE**

| Model | Variable | Coefficient estimate | P-value |
| --- | --- | --- | --- |
| TOD, DD | Time of day | -0.014 | 0.69 |
|  | Dialysis day | 0.14 | 0.04 |
| TOD, TOD squared, DD | Time of day | 0.11 | 0.59 |
|  | Time of day squared | -0.02 | <0.0001 |
|  | Dialysis day | 0.14 | 0.03 |
| TOD, TOD squared, DD, TOD * DD interaction term | Time of day | 0.10 | 0.61 |
|  | Time of day squared | -0.02 | 0.51 |
|  | Dialysis day | 0.10 | 0.50 |
|  | Time of day*dialysis day | 0.01 | 0.80 |
| TOD, TOD squared, DD, TOD * DD interaction term, TOD * DD squared interaction term | Time of day | 0.11 | 0.65 |
|  | Time of day squared | -0.03 | 0.58 |
|  | Dialysis day | 0.13 | 0.72 |
|  | Time of day*dialysis day | -0.009 | 0.98 |
|  | Time of day squared*dialysis day | 0.005 | 0.94 |

eTables 2.

Alternative Factor rotations with respective factor loadings.

|  | **Rotation: Oblimin** | |  |  |
| --- | --- | --- | --- | --- |
|  | POSITIVE MOOD | NEGATIVE MOOD | FATIGUE/SLEEPINESS | ALERT COGNITION |
| Alert | 0.85 | -0.11 | 0.05 | 0.21 |
| Weary | -0.05 | 0.27 | 0.52 | 0.14 |
| Anxious | -0.01 | 0.92 | -0.02 | 0.15 |
| Stressed | 0.04 | 0.86 | 0.12 | -0.07 |
| Tense | 0.03 | 0.90 | 0.08 | -0.14 |
| Sad | -0.01 | 0.86 | -0.03 | -0.01 |
| Irritable | 0.07 | 0.77 | 0.15 | 0.00 |
| Relaxed | 0.82 | 0.19 | -0.12 | -0.11 |
| Energetic | 0.77 | -0.23 | 0.32 | -0.12 |
| Calm | 0.84 | 0.19 | -0.14 | -0.06 |
| Happy | 0.86 | 0.11 | -0.07 | -0.12 |
| Efficient | 0.84 | -0.08 | 0.14 | -0.05 |
| Forgetful | 0.01 | 0.59 | 0.19 | 0.19 |
| Clear-headed | 0.86 | 0.01 | -0.06 | 0.22 |
| Fatigued | -0.03 | 0.11 | 0.81 | -0.01 |
| Sleepy | 0.00 | 0.04 | 0.85 | -0.01 |
| Exhausted | 0.00 | 0.11 | 0.88 | -0.01 |
| Concentrate | 0.22 | 0.20 | 0.31 | 0.25 |
| Effort | 0.23 | 0.08 | 0.31 | 0.12 |
|  |  |  |  |  |

|  | **Rotation: quartimin** | |  |  |
| --- | --- | --- | --- | --- |
|  | POSITIVE MOOD | NEGATIVE MOOD | FATIGUE/SLEEPINESS | ALERT COGNITION |
| Alert | 0.85 | -0.11 | 0.05 | 0.21 |
| Weary | -0.05 | 0.27 | 0.52 | 0.14 |
| Anxious | -0.01 | 0.92 | -0.02 | 0.15 |
| Stressed | 0.04 | 0.86 | 0.12 | -0.07 |
| Tense | 0.03 | 0.90 | 0.08 | -0.14 |
| Sad | -0.01 | 0.86 | -0.03 | -0.01 |
| Irritable | 0.07 | 0.77 | 0.15 | 0.00 |
| Relaxed | 0.82 | 0.19 | -0.12 | -0.11 |
| Energetic | 0.77 | -0.23 | 0.32 | -0.12 |
| Calm | 0.84 | 0.19 | -0.14 | -0.06 |
| Happy | 0.86 | 0.11 | -0.07 | -0.12 |
| Efficient | 0.84 | -0.08 | 0.14 | -0.05 |
| Forgetful | 0.01 | 0.59 | 0.19 | 0.19 |
| Clear-headed | 0.86 | 0.01 | -0.06 | 0.22 |
| Fatigued | -0.03 | 0.11 | 0.81 | -0.01 |
| Sleepy | 0.00 | 0.04 | 0.85 | -0.01 |
| Exhausted | 0.00 | 0.11 | 0.88 | -0.01 |
| Concentrate | 0.22 | 0.20 | 0.31 | 0.25 |
| Effort | 0.23 | 0.08 | 0.31 | 0.12 |
|  |  |  |  |  |

|  | **Rotation: simplimax** | | |  |
| --- | --- | --- | --- | --- |
|  | POSITIVE MOOD | NEGATIVE MOOD | FATIGUE/SLEEPINESS | ALERT COGNITION |
| Alert | 0.68 | 0.47 | 0.02 | 0.31 |
| Weary | -0.35 | 0.52 | 0.36 | 0.15 |
| Anxious | -0.45 | 0.78 | -0.14 | 0.17 |
| Stressed | -0.47 | 0.86 | -0.05 | -0.05 |
| Tense | -0.49 | 0.86 | -0.09 | -0.12 |
| Sad | -0.43 | 0.73 | -0.15 | 0.01 |
| Irritable | -0.40 | 0.81 | -0.01 | 0.03 |
| Relaxed | 0.53 | 0.63 | -0.18 | -0.02 |
| Energetic | 0.54 | 0.51 | 0.22 | -0.04 |
| Calm | 0.55 | 0.63 | -0.19 | 0.03 |
| Happy | 0.58 | 0.62 | -0.13 | -0.03 |
| Efficient | 0.59 | 0.57 | 0.06 | 0.05 |
| Forgetful | -0.34 | 0.62 | 0.06 | 0.21 |
| Clear-headed | 0.67 | 0.52 | -0.08 | 0.32 |
| Fatigued | -0.38 | 0.58 | 0.59 | 0.00 |
| Sleepy | -0.33 | 0.56 | 0.62 | 0.01 |
| Exhausted | -0.38 | 0.64 | 0.64 | 0.00 |
| Concentrate | -0.02 | 0.50 | 0.21 | 0.29 |
| Effort | 0.03 | 0.40 | 0.22 | 0.15 |
|  |  |  |  |  |

|  | **Rotation: bentlerQ** | |  |  |
| --- | --- | --- | --- | --- |
|  | POSITIVE MOOD | NEGATIVE MOOD | FATIGUE/SLEEPINESS | ALERT COGNITION |
| Alert | 0.24 | -0.18 | -0.03 | 0.70 |
| Weary | -0.23 | 0.22 | 0.47 | 0.27 |
| Anxious | -0.24 | 0.87 | -0.07 | 0.29 |
| Stressed | 0.17 | 0.89 | 0.13 | -0.12 |
| Tense | 0.28 | 0.95 | 0.10 | -0.26 |
| Sad | 0.02 | 0.86 | -0.04 | -0.02 |
| Irritable | 0.07 | 0.77 | 0.14 | 0.04 |
| Relaxed | 0.76 | 0.24 | -0.12 | 0.06 |
| Energetic | 0.77 | -0.18 | 0.34 | 0.03 |
| Calm | 0.69 | 0.22 | -0.14 | 0.16 |
| Happy | 0.82 | 0.16 | -0.06 | 0.05 |
| Efficient | 0.68 | -0.05 | 0.13 | 0.20 |
| Forgetful | -0.29 | 0.52 | 0.12 | 0.37 |
| Clear-headed | 0.24 | -0.05 | -0.14 | 0.71 |
| Fatigued | 0.04 | 0.10 | 0.81 | 0.00 |
| Sleepy | 0.07 | 0.03 | 0.84 | 0.02 |
| Exhausted | 0.07 | 0.10 | 0.88 | 0.01 |
| Concentrate | -0.25 | 0.11 | 0.23 | 0.58 |
| Effort | -0.02 | 0.04 | 0.27 | 0.32 |
|  |  |  |  |  |

|  | **Rotation: geominQ** | |  |  |
| --- | --- | --- | --- | --- |
|  | POSITIVE MOOD | NEGATIVE MOOD | FATIGUE/SLEEPINESS | ALERT COGNITION |
| Alert | 0.79 | -0.10 | 0.07 | 0.31 |
| Weary | -0.07 | 0.19 | 0.51 | 0.16 |
| Anxious | -0.05 | 0.86 | -0.03 | 0.20 |
| Stressed | 0.03 | 0.86 | 0.16 | -0.03 |
| Tense | 0.03 | 0.92 | 0.12 | -0.11 |
| Sad | -0.02 | 0.84 | -0.01 | 0.03 |
| Irritable | 0.05 | 0.74 | 0.18 | 0.04 |
| Relaxed | 0.80 | 0.29 | -0.06 | -0.03 |
| Energetic | 0.76 | -0.14 | 0.38 | -0.06 |
| Calm | 0.81 | 0.28 | -0.08 | 0.03 |
| Happy | 0.85 | 0.21 | 0.00 | -0.04 |
| Efficient | 0.82 | 0.00 | 0.20 | 0.03 |
| Forgetful | -0.03 | 0.51 | 0.18 | 0.23 |
| Clear-headed | 0.80 | 0.03 | -0.04 | 0.32 |
| Fatigued | -0.03 | 0.06 | 0.84 | 0.00 |
| Sleepy | 0.01 | -0.01 | 0.87 | 0.01 |
| Exhausted | 0.00 | 0.06 | 0.91 | 0.00 |
| Concentrate | 0.18 | 0.13 | 0.30 | 0.31 |
| Effort | 0.20 | 0.05 | 0.32 | 0.16 |
|  |  |  |  |  |

|  | **Rotation: biquartimin** | | |  |
| --- | --- | --- | --- | --- |
|  | NEGATIVE MOOD | POSITIVE MOOD | FATIGUE/SLEEPINESS | ALERT COGNITION |
| Alert | 0.73 | -0.30 | -0.18 | 0.21 |
| Weary | 0.39 | 0.17 | 0.48 | 0.13 |
| Anxious | 0.51 | 0.76 | -0.03 | 0.16 |
| Stressed | 0.59 | 0.72 | 0.05 | -0.07 |
| Tense | 0.58 | 0.76 | 0.00 | -0.13 |
| Sad | 0.47 | 0.72 | -0.06 | 0.00 |
| Irritable | 0.58 | 0.61 | 0.08 | 0.01 |
| Relaxed | 0.78 | 0.02 | -0.38 | -0.10 |
| Energetic | 0.73 | -0.36 | 0.04 | -0.13 |
| Calm | 0.79 | 0.01 | -0.39 | -0.05 |
| Happy | 0.80 | -0.06 | -0.35 | -0.12 |
| Efficient | 0.78 | -0.24 | -0.13 | -0.05 |
| Forgetful | 0.45 | 0.45 | 0.16 | 0.19 |
| Clear-headed | 0.75 | -0.19 | -0.29 | 0.22 |
| Fatigued | 0.47 | 0.01 | 0.73 | -0.02 |
| Sleepy | 0.48 | -0.05 | 0.75 | -0.02 |
| Exhausted | 0.53 | 0.00 | 0.78 | -0.02 |
| Concentrate | 0.49 | 0.06 | 0.23 | 0.25 |
| Effort | 0.42 | -0.03 | 0.22 | 0.12 |
|  |  |  |  |  |

|  | **Rotation: varimax** | |  |  |
| --- | --- | --- | --- | --- |
|  | POSITIVE MOOD | NEGATIVE MOOD | FATIGUE/SLEEPINESS | ALERT COGNITION |
| Alert | 0.82 | -0.01 | 0.10 | 0.24 |
| Weary | 0.01 | 0.40 | 0.58 | 0.12 |
| Anxious | 0.09 | 0.87 | 0.25 | 0.15 |
| Stressed | 0.15 | 0.87 | 0.35 | -0.07 |
| Tense | 0.14 | 0.89 | 0.31 | -0.13 |
| Sad | 0.09 | 0.81 | 0.21 | 0.00 |
| Irritable | 0.17 | 0.78 | 0.36 | 0.01 |
| Relaxed | 0.83 | 0.23 | -0.02 | -0.07 |
| Energetic | 0.76 | -0.05 | 0.29 | -0.11 |
| Calm | 0.85 | 0.23 | -0.02 | -0.02 |
| Happy | 0.87 | 0.17 | 0.01 | -0.08 |
| Efficient | 0.83 | 0.05 | 0.17 | -0.02 |
| Forgetful | 0.08 | 0.61 | 0.36 | 0.18 |
| Clear-headed | 0.84 | 0.08 | 0.03 | 0.25 |
| Fatigued | 0.03 | 0.33 | 0.80 | -0.04 |
| Sleepy | 0.06 | 0.28 | 0.82 | -0.04 |
| Exhausted | 0.07 | 0.36 | 0.87 | -0.04 |
| Concentrate | 0.25 | 0.30 | 0.39 | 0.25 |
| Effort | 0.25 | 0.19 | 0.35 | 0.11 |
|  |  |  |  |  |

|  | **Rotation: quartimax** | |  |  |
| --- | --- | --- | --- | --- |
|  | NEGATIVE MOOD | POSITIVE MOOD | FATIGUE/SLEEPINESS | ALERT COGNITION |
| Alert | 0.03 | 0.83 | 0.07 | 0.22 |
| Weary | 0.56 | 0.01 | 0.42 | 0.13 |
| Anxious | 0.91 | 0.09 | -0.04 | 0.14 |
| Stressed | 0.93 | 0.14 | 0.08 | -0.08 |
| Tense | 0.94 | 0.13 | 0.04 | -0.15 |
| Sad | 0.83 | 0.09 | -0.05 | -0.01 |
| Irritable | 0.86 | 0.17 | 0.11 | 0.00 |
| Relaxed | 0.22 | 0.83 | -0.09 | -0.10 |
| Energetic | 0.05 | 0.76 | 0.28 | -0.12 |
| Calm | 0.22 | 0.84 | -0.10 | -0.06 |
| Happy | 0.17 | 0.87 | -0.05 | -0.12 |
| Efficient | 0.11 | 0.83 | 0.13 | -0.04 |
| Forgetful | 0.70 | 0.08 | 0.14 | 0.18 |
| Clear-headed | 0.10 | 0.85 | -0.02 | 0.22 |
| Fatigued | 0.56 | 0.04 | 0.66 | -0.01 |
| Sleepy | 0.52 | 0.07 | 0.70 | -0.01 |
| Exhausted | 0.61 | 0.07 | 0.72 | -0.01 |
| Concentrate | 0.41 | 0.26 | 0.26 | 0.25 |
| Effort | 0.29 | 0.25 | 0.26 | 0.12 |
|  |  |  |  |  |

|  | **Rotation: bentlerT** | |  |  |
| --- | --- | --- | --- | --- |
|  | POSITIVE MOOD | NEGATIVE MOOD | FATIGUE/SLEEPINESS | ALERT COGNITION |
| Alert | 0.80 | -0.05 | -0.01 | 0.30 |
| Weary | 0.08 | 0.46 | 0.51 | 0.19 |
| Anxious | 0.15 | 0.88 | 0.12 | 0.20 |
| Stressed | 0.23 | 0.89 | 0.24 | 0.00 |
| Tense | 0.23 | 0.91 | 0.21 | -0.07 |
| Sad | 0.15 | 0.82 | 0.10 | 0.04 |
| Irritable | 0.24 | 0.80 | 0.25 | 0.07 |
| Relaxed | 0.84 | 0.18 | -0.12 | -0.02 |
| Energetic | 0.79 | -0.06 | 0.22 | -0.03 |
| Calm | 0.85 | 0.17 | -0.14 | 0.03 |
| Happy | 0.88 | 0.12 | -0.09 | -0.03 |
| Efficient | 0.84 | 0.02 | 0.07 | 0.05 |
| Forgetful | 0.13 | 0.64 | 0.25 | 0.24 |
| Clear-headed | 0.82 | 0.03 | -0.10 | 0.30 |
| Fatigued | 0.13 | 0.41 | 0.75 | 0.06 |
| Sleepy | 0.16 | 0.37 | 0.77 | 0.07 |
| Exhausted | 0.18 | 0.45 | 0.81 | 0.07 |
| Concentrate | 0.29 | 0.32 | 0.30 | 0.31 |
| Effort | 0.28 | 0.21 | 0.28 | 0.17 |
|  |  |  |  |  |

|  | **Rotation: geominT** | |  |  |
| --- | --- | --- | --- | --- |
|  | NEGATIVE MOOD | POSITIVE MOOD | FATIGUE/SLEEPINESS | ALERT COGNITION |
| Alert | 0.05 | 0.80 | 0.11 | 0.29 |
| Weary | 0.58 | -0.03 | 0.39 | 0.13 |
| Anxious | 0.91 | 0.07 | -0.08 | 0.13 |
| Stressed | 0.94 | 0.13 | 0.03 | -0.08 |
| Tense | 0.94 | 0.13 | -0.01 | -0.15 |
| Sad | 0.83 | 0.08 | -0.09 | -0.02 |
| Irritable | 0.86 | 0.15 | 0.07 | 0.00 |
| Relaxed | 0.23 | 0.84 | -0.06 | -0.03 |
| Energetic | 0.07 | 0.75 | 0.32 | -0.04 |
| Calm | 0.22 | 0.85 | -0.07 | 0.01 |
| Happy | 0.18 | 0.87 | -0.01 | -0.04 |
| Efficient | 0.13 | 0.82 | 0.17 | 0.03 |
| Forgetful | 0.71 | 0.05 | 0.10 | 0.18 |
| Clear-headed | 0.11 | 0.83 | 0.01 | 0.29 |
| Fatigued | 0.60 | -0.01 | 0.63 | 0.00 |
| Sleepy | 0.56 | 0.03 | 0.67 | 0.00 |
| Exhausted | 0.65 | 0.03 | 0.69 | 0.00 |
| Concentrate | 0.43 | 0.22 | 0.25 | 0.27 |
| Effort | 0.31 | 0.22 | 0.26 | 0.14 |

**eTable 3**. Association of Fatigue-Sleepiness-Exhaustion composite score and time of day and dialysis day using linear mixed regression modeling with quadratic term

| **Variable** | **Coefficient (95% CI)** | **P-value** |
| --- | --- | --- |
| Time of day | -0.81 (-1.28, -0.37) | <0.001 |
| Time of day squared | 0.23 (0.14, 0.32) | <0.001 |
| Dialysis day | 0.20 (0.02, 0.38) | 0.005 |

**eTable 4**. Adjusted association of factor analysis derived FSE score and time of day, time of day squared, and dialysis day.

| **Model** | **Variable** | **Coefficient estimate (95% CI)** | **P-value** |
| --- | --- | --- | --- |
| Unadjusted | Time of day | -0.50 (-0.78, -0.23) | <0.001 |
|  | Time of day squared | 0.14 (0.09, 0.20) | <0.001 |
|  | Dialysis day | 0.13 (0.02, 0.24) | 0.02 |
| Adjusted for demographics* | Age (per year) | -0.02 (-0.03, -0.01) | <0.001 |
|  | Sex (female vs. male) | -0.002 (-0.28, 0.27) | 0.99 |
|  | Race (black vs. non-black) | -0.24 (-0.50, 0.06) | 0.12 |
|  | Time of day | -0.50 (-0.78, -0.24) | 0.001 |
|  | Time of day squared | 0.14 (0.09, 0.20) | <0.001 |
|  | Dialysis day | 0.13 (0.02, 0.24) | 0.02 |
| Demographics + Kt/V | Age (per year) | -0.018 (-0.03, -0.01) | <0.001 |
|  | Sex (female vs. male) | -0.047 (-0.37, 0.29) | 0.81 |
|  | Race (black vs. non-black) | -0.38 (-0.70, -0.03) | 0.04 |
|  | Kt/V | -0.30 (-0.99, 0.45) | 0.45 |
|  | Time of day | -0.47 (-0.79, -0.17) | 0.003 |
|  | Time of day squared | 0.14 (0.08, 0.20) | <0.001 |
|  | Dialysis day | 0.10 (-0.02, 0.23) | 0.10 |

*Serial additional adjustment for diabetes, cardiovascular disease, albumin, hemoglobin, and phosphate did not affect time of day or dialysis day coefficients.
